# Effect of pH on Lipid Oxidation and Anthocyanin Stability in Flaxseed Oil‐in‐Water Emulsions With Black Carrot Extract

**DOI:** 10.1111/1750-3841.70568

**Published:** 2025-09-21

**Authors:** Evelyn Klinger, Hanna Salminen, Karola Bause, Jochen Weiss

**Affiliations:** ^1^ Department of Food Material Science, Institute of Food Science and Biotechnology University of Hohenheim Stuttgart Germany; ^2^ GNT Europa GmbH Aachen Germany

## Abstract

The strong pH sensitivity and the activity toward free radical reactions limit the use of anthocyanins as food colorants in emulsion‐based food applications. The aim of this study was to gain a comprehensive understanding of color stability of anthocyanin‐rich black carrot extracts in ω‐3 fatty acid–rich oil‐in‐water emulsions as a function of pH. The stability of the anthocyanins was influenced by the degree of lipid oxidation, which in turn depended on the pH value. The formation of lipid oxidation products was accelerated in emulsions without added black carrot extract, which is associated with the antioxidant activity of anthocyanins. Furthermore, lipid oxidation increased with decreasing pH due to a higher solubility of catalyzing metals like iron. The anthocyanins showed a pH‐dependent degradation kinetics: pH 2 (*k* = 1.44 h^−1^) > pH 3 (*k* = 0.3 h^−1^) > pH 4 (*k* = 0.06 h^−1^) ≅ pH 6 (*k* = 0.09 h^−1^), which correlated with reduced concentrations of lipid oxidation products. This is contrary to the common behavior of anthocyanins in aqueous solutions, where their stability increases with decreasing pH. The best color stability of the black carrot extract in the oil‐in‐water emulsion was observed at pH 4 instead of pH 6, due to a pH‐dependent deprotonation reaction. Gaining insights into the complex relationship between pH, lipid oxidation, and the color stability of anthocyanin‐rich black carrot extracts in ω‐3 fatty acid–rich emulsions challenges conventional understanding and offers valuable guidance for improving the use of anthocyanins as food colorants in emulsion‐based applications.

**Practical Applications:**

This study provides insight into the stability of anthocyanin‐based natural colorants, such as black carrot extract, in oil‐in‐water emulsions, facilitating their application in lipid‐containing food systems. It provides a more comprehensive understanding of the pH‐dependent degradation mechanisms of anthocyanins, extending current knowledge from aqueous solutions to emulsion‐based conditions.

## 1 Introduction

Edible foods with a high pigment content present a natural alternative to artificial colorants by preserving the appearance of food colors (Kammerer 2024). Anthocyanins, a class of natural pigments widely found in plants, have gained considerable attention in recent years due to their health benefits and a variety of hues with over 600 different structures (Carocho et al. 2015). However, stability issues remain a significant challenge for the use of coloring foods (Kammerer 2024). Certain stabilization mechanisms, such as self‐association and copigmentation including acylation with organic acids, are known to enhance the resistance of anthocyanins to degradation reactions (Dangles and Fenger 2018). Our previous study showed that the anthocyanins in black carrot extract contained a high amount (47%) of acylated anthocyanins, namely sinapic and ferulic derivatives of cyanidin 3‐xylosyl galactosides (Klinger et al. 2024a). Such highly acylated plant extracts are better at inhibiting lipid oxidation in polyunsaturated oil‐in‐water emulsions than hibiscus or chokeberry extracts with low amounts of acylated acids. The presence of acyl groups in anthocyanins contributes to the stabilization of the aglycone via intramolecular π‐stacking interactions (Dangles and Fenger 2018).

Anthocyanins are more stable in acidic solutions due to the presence of the flavylium cation form (Khoo et al. 2017), while a higher pH leads to deprotonation on the ring structure and the formation of neutral quinoid bases and a further deprotonation to blue anionic quinoid bases (Dangles and Fenger 2018). This typically shifts the wavelength of maximum visible absorption by 20–30 nm that is accompanied by a color change from red to purple (Dangles and Fenger 2018).

Furthermore, anthocyanins have been found to be effective in inhibiting the formation of lipid oxidation products (Salminen et al. 2010; Kähkönen and Heinonen 2003; Gan et al. 2012; Yi et al. 2020; Klinger et al. 2024a, 2024b), which can be attributed to the conjugated structure of anthocyanins enabling antioxidant activity through electron delocalization, leading to the formation of stable radical products (Kähkönen and Heinonen 2003). However, this antioxidative mechanism also involves the disruption of the anthocyanin aglycone's conjugated π‐electron system, which is responsible for their characteristic visible color (Dangles and Fenger 2018).

It is known that the oxidative stability of oil‐in‐water emulsions depends, besides the presence of antioxidants, on other factors such as unsaturation of the fatty acids, availability of oxygen, the presence of prooxidants, pH of the water phase, and water activity (Kim et al. 2016; Kim and Choe 2018).

Previous studies on pH‐dependent oxidative stability of oil‐in‐water emulsions have yielded widely varying results (Kim and Choe 2018; Kim et al. 2016; Riisom et al. 1980; Mei, Decker, et al. 1998; Mei, McClements, et al., 1998; Haahr and Jacobsen 2008; Horn et al. 2012; Hu et al. 2003). In several studies, oxidation was faster at pH 7 than at pH 3, for example, in 5% salmon oil‐in‐water emulsions stabilized by sodium dodecyl sulfate (SDS), Tween 20, or dodecyl trimethylammonium bromide (Mancuso et al. 1999), in whey protein–stabilized salmon oil‐in‐water emulsions (Hu et al. 2003), in corn oil‐in‐water emulsions without emulsifier (Kim et al. 2016), and in soybean oil‐in‐water emulsions stabilized by lecithin (Kim and Choe 2018). In contrast, oxidation proceeded faster at low pH in 10% medium chain triglyceride/fish oil‐in‐water (1:1) emulsions stabilized by lecithin, Citrem, Tween 80, or sodium caseinate (Haahr and Jacobsen 2008). Better oxidative stability was also observed at pH 7 than at pH 4.5 in whey protein–stabilized 70% fish oil‐in‐water emulsion (Horn et al. 2012). Further studies reported increasing oxidative stability of safflower oil‐in‐water emulsions stabilized by sodium stearoyl lactylate from pH 6 to 8 (Riisom et al. 1980) and higher stability of SDS‐stabilized oil‐in‐water emulsions at pH 5 compared to pH 3 (Mei, McClements, et al. 1998). The majority of these studies indicated that increasing the pH reduces the solubility of the ubiquitous transition metals in the water phase of the emulsion, thus decreasing the concentration of these prooxidants (Kim et al. 2016) and, in some cases, subsequently leading to a reduction in lipid oxidation reactions (Haahr and Jacobsen 2008; Horn et al. 2012; Riisom et al. 1980; Mei, McClements, et al. 1998).

Polyphenols can exhibit prooxidant or antioxidant effects depending on the pH. For example, a prooxidant effect of hydroxytyrosol and oleuropein was observed in olive oil‐in‐water emulsions stabilized by Tween 20 at pH 3.5 in the presence of iron ions, while both phenolic compounds exhibited antioxidant activity at pH 7.4 (Paiva‐Martins and Gordon 2002). Similarly, Sørensen et al. (2008) found no antioxidant effect at pH 3 but a significant antioxidant activity at pH 7 in 10% oil‐in‐water emulsions (Miglyol/fish oil 1:1) stabilized by Tween 80. Caffeic acid demonstrated similar behavior in the same emulsion system (Sørensen et al. 2008). Anthocyanin‐rich extracts from sweet potatoes also showed increasing antioxidant activity with rising pH, which is attributed to the formation of quinoidal bases chalcones and hemiketals. These forms are less stable and are more prone to react as radical scavengers (Liu et al. 2023; Shi et al. 2021).

Enhanced antioxidant activity at higher pH levels can be attributed to several factors. While lipid oxidation generally decreases at elevated pH, the ionization state of phenolic compounds, their redox potential, the stability of the resulting radicals, and the chelation of metal ions also contribute significantly (Paiva‐Martins and Gordon 2002; Sørensen et al. 2008; Rice‐Evans et al. 1997; Shahidi and Zhong 2010; Roginsky and Lissi 2005; Shahidi et al. 1992). At higher pH, phenolic hydroxyl groups are predominantly ionized, enhancing stability and electron donation, while the lower redox potential increases the antioxidant capacity (Rice‐Evans et al. 1997; Shahidi and Zhong 2010). The stability of phenoxy radicals formed during oxidation is also higher at elevated pH, leading to more effective interruption of radical chain reactions (Roginsky and Lissi 2005), and increased metal ion chelation at higher pH reduces their prooxidant activity (Shahidi et al. 1992). As no studies have examined anthocyanins in oil‐in‐water emulsions across a range of pH values, this study investigated the stability of anthocyanins from black carrot extract in flaxseed oil‐in‐water emulsions stabilized by SDS at pH 2, 3, 4, and 6. Based on previous literature (Mei, McClements, et al. 1998), we hypothesized that lower pH accelerates lipid oxidation and that the increased formation of lipid oxidation products leads to faster degradation of anthocyanins as they act as free radical scavengers. Nevertheless, the increasing pH can lead to faster degradation of anthocyanins through deprotonation reactions.

## 2 Materials and Methods

### 2.1 Materials

Coloring foods from black carrot (*Daucus carota* L. ssp. *sativus* var. *atrorubens* Alef; liquid) were provided by GNT Europa GmbH (Aachen, Germany). Black carrots were processed nonselectively by water extraction, solid–liquid separation, enzymatic catalysis, and evaporation. The black carrot extract contained 0.0132% of anthocyanins (35.3% of sinapic acid derivatives of cyanidin 3‐xylosylglucosyl galactosides, 33.2% of cyanidin 3‐xylosyl galactosides, 12.9% of cyanidin 3‐xylosylglucosyl galactosides, 10.4% of ferulic acid derivatives of cyanidin 3‐xylosylglucosyl galactosides, and 1.4% of p‐coumaric acid derivatives of cyanidin 3‐xylosylglucosyl galactosides) (Klinger et al. 2024a). The native flaxseed oil (76% α‐linolenic acid, 9% linoleic acid, 4% oleic acid, and 1.5% stearic acid) was purchased from Ölmühle Ditzingen GmbH & Co. KG (Ditzingen, Germany). Acetonitrile (>99.9%), ammonium thiocyanate (>99%), barium chloride (>99%), 2‐butanone (98%), citric acid (>99.5%), 2,2‐diphenyl‐1‐picrylhydrazyl (DPPH), 2‐ethylfuran (98%), ferrous sulfate (>99%), Folin–Ciocalteu reagent, formic acid (>98%), gallic acid (>99%), hexanal (98%), hydrochloric acid (32%), methyl pentadecanoate (>99.5%), phosphoric acid (85%), propanal (97%), sodium azide (>99%), sodium carbonate (>99%), sodium citrate dihydrate (>99%), and SDS (>99%) were purchased from Merck (Darmstadt, Germany). 1‐Butanol, hexane, isooctane, methanol, and 1‐propanol were obtained from Carl Roth GmbH & Co. KG (Karlsruhe, Germany) and had purities ≥99.5%. Cyanidin 3‐glucoside standard was purchased from Extrasynthese (Genay, France). Double distilled water was used throughout the study.

### 2.2 Sample Preparation

The water phase of the oil‐in‐water emulsion consisted of 0.1% (w/w) (= 3.47 mM) SDS dissolved in a 10‐mM citrate buffer adjusted to pH 2, 3, 4, and 6. SDS was selected as surfactant because it is effective in oil‐in‐water emulsions over a broad pH range and it does not induce perceptible color shifts in emulsions like other emulsifiers such as lecithin or saponins (Uluata et al. 2015). Although recognized as safe (GRAS) as a food additive by the Food and Drug Administration (FDA 2025), its use as an emulsifier is restricted in food applications. Therefore, SDS served as a model surfactant to study anthocyanin behavior in emulsions.

Flaxseed oil (1% w/w) was added to the surfactant solution and homogenized by a high‐shear (15,000 rpm) Silent Crusher M homogenizer (Heidolph Instruments GmbH and Co. KG; Schwabach, Germany) for 2 min. To further reduce the emulsion droplet size, a high‐pressure homogenization was carried out using an LM10 Microfluidizer equipped with a G10Z interaction chamber with a diameter of 87 µm (Microfluidics Corporation, Westwood, MA, USA) at 1500 bar with three cycles. After homogenization, 0.73 g L^−1^ black carrot extract was added to the emulsion. An emulsion without added black carrot extract was used as reference sample. Transparent glass vials (10 mL, 18 × 50 mm; VWR International, Radnor, PA, USA) were used to store all prepared samples. The vials were sealed with plastic caps and placed in a dark climate cabinet maintained at 35°C. For GC–MS analysis, 1 mL of the samples was stored in brown glass headspace vials (20 mL, 23 × 75 mm) sealed with magnetic screw caps (Agilent Technologies GmbH & Co. KG, Waldbronn, Germany) under the same conditions as the other sample vials.

### 2.3 Droplet Size

The mean volume diameter (*d*_4,3_) of the oil‐in‐water emulsions was determined using a static light scattering device (Horiba LA‐950; Retsch Technology, Haan, Germany). To prevent multiple scattering effects, the samples were diluted with the respective citrate buffer at pH 2, 3, 4, or 6. The hydrodynamic diameter (*d*_h_) of the emulsions was measured using a dynamic light scattering device (Nano‐Zetasizer; Malvern Instruments Ltd., Worcestershire, UK). The samples were directly filled into a folded capillary cell and measured with a laser wavelength of 633 nm at 25°C. Backscattered light was detected at an angle of 173°. For both methods, the refractive indices were set to 1.48 for the flaxseed oil and 1.33 for the aqueous citrate buffer phase.

### 2.4 *ζ*‐Potential

A laser doppler electrophoresis instrument (Nano‐Zetasizer; Malvern Instruments Ltd.) was utilized to measure the net charge of the SDS‐stabilized oil‐in‐water emulsion droplets in the aqueous buffer solution at pH 2–6 at 25°C. The samples were measured without further dilutions.

### 2.5 Color Measurements

First, to investigate the influence of SDS addition on the spectroscopic properties of black carrot extract, 0.73 g L^−1^ black carrot extract was added to 10 mM citrate buffer at pH 2–6 with and without added 0.1% SDS. The absorbance spectra were recorded across a wavelength range of 380–780 nm using a Lambda 750s spectrophotometer equipped with a 60‐mm integrating sphere (Perkin Elmer, Waltham, MA, USA).

Second, the emulsions at pH 3, 4, and 6 were adjusted to pH 2 by 0.5 M HCl solution. This was followed by a hexane extraction, as the turbidity of the emulsions prevented direct measurement of the absorbance. For this, 6 mL of emulsion was vortexed with 3 mL of hexane for 60 s and centrifugated at 15,000 × *g* for 15 min at 20°C. The solvent phase was removed, and the water phase containing the black carrot extract was transferred into polystyrene macro cuvettes (VWR International, Radnor, PA, USA). Due to the hydrophilic properties of the anthocyanins, no color remained in the solvent phase. No solvent extraction was performed for the black carrot extract solution samples with or without added SDS. The absorbance spectra were then measured as described above. To ensure that no residual turbidity affected the results, a correction was made by subtracting the absorbance at 750 nm from the absorbance at the maximum wavelength of the black carrot anthocyanins at 522 nm. The concentration of anthocyanins was calculated according to a calibration curve of cyanidin 3‐glucoside dissolved in 10 mM citrate buffer at pH 2.

The concentration of anthocyanins (*C_t_*, µM) in the emulsion samples at pH 2–6 was modeled as a function of time (Equation 1):

(1) $$C_{t}=C_{0}\times e^{-kt},$$

where *C*_0_ was the anthocyanin concentration (µM) at *t* = 0, *k* was the reaction rate constant (day^−1^), and *t* was the time (days). The half‐life time (*t*_1/2_, days) (Equation 2) denotes the time at which the concentration was half of the initial concentration:

(2) $$t_{1/2}=\frac{\ln2}{k}.$$

Rate constant *k* was obtained from fitted values, which can be found in Table S1.

Additionally, the colorimetric values L*, C*, and *h*° were obtained from the absorbance spectra according to CIELAB 1931 with an observer angle of 2° and illuminant C. The colors were extracted using GIMP version 2.10.24 (available at https://gimp.org/). The color differences (Δ*E*_00_) between the sample colors at 0 and 7 days were determined using the CIE Δ*E*_2000_ formula, the most recent refinement of the Δ*E* calculation.

### 2.6 Lipid Oxidation

#### 2.6.1 Lipid Hydroperoxides

The formation of lipid hydroperoxides was determined using a method adapted from Shantha and Decker (1994) with some modifications. In short, 0.3 mL of the emulsion was mixed with 1.5 mL of an isooctane:2‐propanol solution (3:1, v/v) by vortexing for 10 s, three times. The mixture was then centrifuged at 1000 × *g* for 3 min. A 100‐µL aliquot of the resulting organic phase was transferred and combined with 2.8 mL of a methanol:1‐butanol solution (2:1, v/v). Subsequently, 30 µL of a thiocyanate‐ferrous reagent was added. The reagent was prepared by mixing equal volumes of 0.132 BaCl_2_ and 0.144 mM FeSO_4_, centrifuging, and then combining equal volumes of the resulting clear supernatant with 3.97 M ammonium thiocyanate. After 20 min of incubation, the absorbance at 510 nm was measured using a Mettler Toledo UV/VIS‐UV7 spectrophotometer (Mettler Toledo GmbH, Giessen, Germany). Lipid hydroperoxide concentration was determined by a six‐point calibration curve of cumene hydroperoxide in methanol:1‐butanol (2:1, v/v).

#### 2.6.2 Volatiles

The volatile secondary oxidation products propanal and hexanal were determined as described previously (Klinger et al. 2024a) by using a gas chromatograph (Intuvo 9000 GC) coupled with a 5977B single quadrupole mass spectrometer (Agilent Technologies, Waldbronn, Germany) and a PAL3 autosampler. The GC was equipped with a DB‐HeavyWAX Intuvo capillary column (30 m × 0.25 mm internal diameter and 0.5 µm film thickness) and an MMI guard chip (set to 65°C). Emulsion samples (1.0 mL) were transferred into 20‐mL amber headspace vials and sealed with magnetic screw caps containing PTFE/silicone septa. Prior to analysis, vials were incubated at 55°C for 15 min. A headspace volume of 1 mL was injected with an injection flow rate of 10 mL min^−1^, and the inlet temperature was set to 260°C with a split ration of 5:1. Helium was used as carrier gas. The oven temperature program was as follows: 40°C for 3 min, ramped to 80°C at 5°C min^−1^, to 120°C at 7°C min^−1^, to 180°C at 15°C min^−1^, and finally to 240°C at 30°C min^−1^, held for 3 min. The MS operated in electron ionization mode (70 eV), with source and transfer line temperatures set to 230°C. Spectra were acquired in scan mode (*m*/*z* 25–250, scan speed 781 u s^−1^, scan cycle 313 ms) with a solvent delay of 2.5 min. Compound identification was based on major qualifier ions and comparison with the NIST 2020 mass spectral library. A six‐point external calibration curve (propanal: 0.02–2.8 mM, hexanal: 0.01–0.16 mM) was used for the quantification.

#### 2.6.3 Kinetic Modeling

The kinetics of lipid hydroperoxides and volatiles were fitted using Excel Solver (Microsoft, Redmond, WA, USA) with a sigmoidal model (Equation 3) that comprises the lipid oxidation product concentration *c* (µM), the initial lipid oxidation product concentration *c*_0_ (µM) at *t* = 0, the maximum lipid oxidation product concentration *c*_max_ (µM), the rate constant *k* (day^−1^), and the time *t* (days):

(3) $$c=\frac{c_{\max}}{1+\left(\frac{c_{\max}-c_{0}}{c_{0}}\right)\times e^{-kt}}.$$

The second derivative of the function reveals the turning point coordinates, where the rate of accumulation of oxidation products reaches a maximum value during the propagation phase of lipid oxidation. If the inflection tangent resulting from the slope of the sigmoidal function (Equation 3) and the coordinates of the inflection point intersected with the *x*‐axis, the value for the induction phase *t*_ind_ (days) was obtained.

### 2.7 Statistics

Each emulsion was prepared two times and measured at least in duplicates. All results are expressed as means with standard deviations (Excel; Microsoft). Statistical differences were evaluated using a one‐way ANOVA with a Tukey's post hoc test (*p* = 0.05) using SPSS Statistics 29 (IBM, Armonk, NY, USA).

## 3 Results and Discussion

### 3.1 Emulsion Properties

The flaxseed oil‐in‐water emulsions were nanosized at all pH values (*d*_h_ = 135–154 nm, *d*_4,3_ = 118–209 nm) and remained stable during the 21 days of storage (Table 1). This was demonstrated by the low polydispersity (PDI = 0.16–0.22) (Table 1) and particle size distribution results (data not shown), indicating monomodally distributed emulsion droplets. A slight increase (*p* < 0.05) in the mean hydrodynamic diameter (*d*_h_) was observed for emulsions at pH 2 and at pH 3 (with extract) after 21 days of storage. However, no significant changes in *d*_4,3_‐value were observed between pH 2 and 6 (Table 1). The slightly higher net *ζ*‐potential values in the emulsions with the added black carrot extract at pH 2–4 (Table 1) can be explained by the presence of flavylium cations in the black carrot extract and electrostatic binding to the negatively charged surface of the emulsion droplets (Dangles and Fenger 2018; Yi et al. 2020). At pH 6, on the other hand, the flavylium cation is fully deprotonated, and neutral or anionic quinoid bases are formed (Dangles and Fenger 2018), which explains that the black carrot extract has a lesser impact on the *ζ*‐potential at pH 6 (Table 1).

**TABLE 1:** Hydrodynamic diameter (*d*_h_), volume‐based mean particle diameter (*d*_4,3_), polydispersity index (PDI), and *ζ*‐potential of flaxseed oil‐in‐water emulsion (1% w/w oil, 0.1% w/w SDS in 10 mM citrate buffer) at different pH values with or without added black carrot extract (0.73 g L^−1^) during 21 days of storage at 35°C in the dark.

*c*_extract_ (g L^−1^)	*d*_h_ (nm)		*d*_4,3_ (nm)		PDI (–)		*ζ* (mV)	
	Day 0	Day 21	Day 0	Day 21	Day 0	Day 21	Day 0	Day 21
pH 2								
0	136 ± 1^a^	154 ± 3^d^	197 ± 3^b^	209 ± 8^b^	0.17 ± 0.01^a^	0.16 ± 0.02^a^	−97 ± 8^bc^	−101 ± 6^a^
0.73	140 ± 3^a^	149 ± 5^cd^	194 ± 4^b^	198 ± 7^b^	0.18 ± 0.02^a^	0.16 ± 0.01^a^	−70 ± 3^e^	−84 ± 6^bc^
pH 3								
0	135 ± 2^a^	135 ± 2^ab^	120 ± 5^a^	131 ± 3^a^	0.16 ± 0.00^a^	0.16 ± 0.01^a^	−80 ± 8^d^	−80 ± 8^bc^
0.73	139 ± 2^a^	149 ± 4^bc^	143 ± 4^a^	140 ± 3^a^	0.21 ± 0.01^ab^	0.17 ± 0.01^a^	−73 ± 3^de^	−82 ± 6^c^
pH 4								
0	135 ± 2^a^	140 ± 2^a^	118 ± 1^a^	118 ± 1^a^	0.18 ± 0.01^ab^	0.20 ± 0.02^ab^	−108 ± 4^a^	−100 ± 3^a^
0.73	140 ± 2^a^	144 ± 8^abc^	142 ± 37^a^	152 ± 4^a^	0.18 ± 0.01^ab^	0.22 ± 0.05^b^	−93 ± 3^c^	−84 ± 3^c^
pH 6								
0	141 ± 2^a^	139 ± 2^a^	194 ± 2^b^	206 ± 9^b^	0.18 ± 0.01^ab^	0.18 ± 0.02^a^	−93 ± 3^ab^	−102 ± 5^a^
0.73	139 ± 2^a^	138 ± 2^a^	194 ± 4^b^	191 ± 4^b^	0.20 ± 0.01^ab^	0.18 ± 0.01^a^	−103 ± 5^abc^	−93 ± 6^ab^

*Note*: Different small letters within each parameter and day denote a significant difference according to Tukey post hoc test (*p* < 0.05).

### 3.2 Color Properties of Black Carrot Extract

**FIGURE 1:**
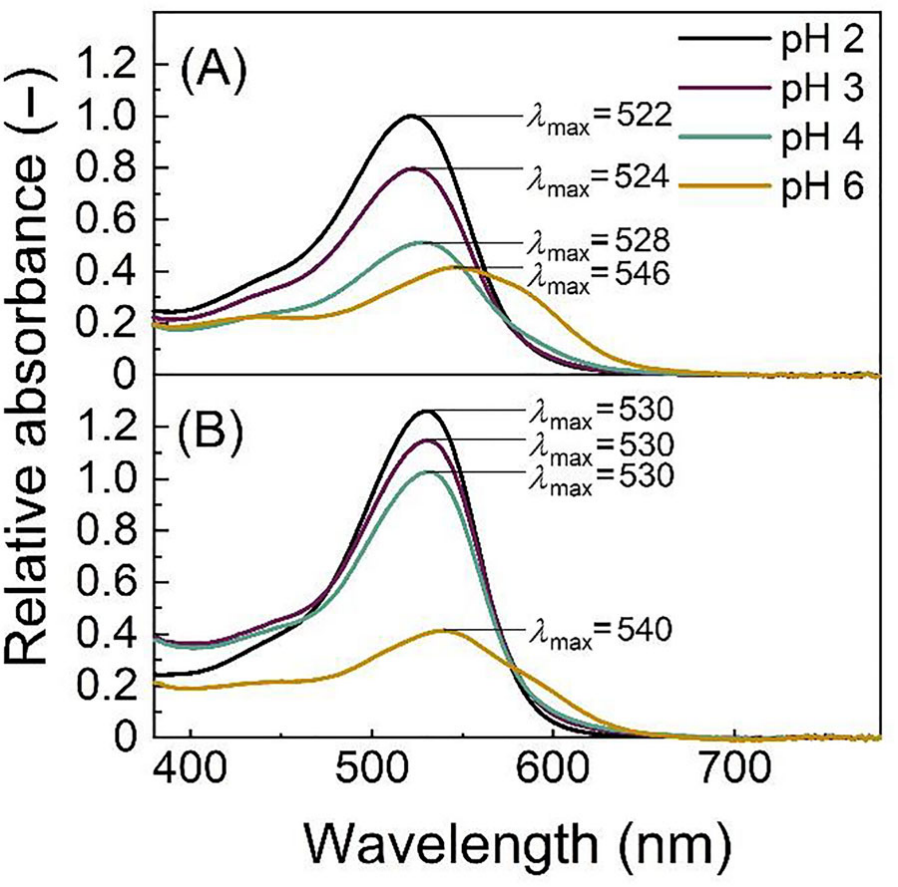
Impact of pH on the absorbance spectra of aqueous black carrot extract (0.73 g L^−1^) in 10 mM citrate buffer at different pH values without SDS (A) and with 0.1% (w/w) SDS (B).

To determine the effect of pH and SDS on the spectroscopic properties of the black carrot extract, the absorbance spectra of the black carrot extract (0.73 g L^−1^) in 10 mM citrate buffer at pH 2–6 with and without added SDS (0.1%, w/w) was measured (Figure 1).

The maximum absorbance of the black carrot extract at pH 2 was detected at 522 nm. Increasing the pH to 3, 4, and 6 resulted in a bathochromic shift, with maximum wavelengths of 524, 528, and 546 nm, respectively (Figure 1A). Furthermore, a hypochromic shift was observed upon increasing the pH. The red flavylium cation, which predominates at pH 2, undergoes conversion into a neutral quinonoid base, followed by transformation into an anionic quinonoid base (pH 2–4). At pH 6, theoretically, colorless species can be observed, explaining the decrease in absorbance with increasing pH (Dangles and Fenger 2018; Castañeda‐Ovando et al. 2009). However, intramolecular co‐pigmentation, occurring in acylated anthocyanins, protects the molecules from hydration reactions, resulting in residual color even at higher pH values. This is in line with a previous study on the spectral data of black carrots at different pH values (Bay Yılmaz and Türker 2023). Investigations at pH >6, relevant for certain food applications such as some dairy products, were not carried out in this study due to the rapid conversion of black carrot anthocyanins into unstable hemiketal and chalcone forms above these pH values (Stintzing et al. 2002; Castañeda‐Ovando et al. 2009). Additionally, the reduced absorbance in the visible spectrum makes spectrophotometric monitoring of color degradation challenging (Dangles and Fenger 2018).

Addition of SDS resulted in further bathochromic shifts of 8 nm at pH 2, 6 nm at pH 3, and 2 nm at pH 4, resulting in *λ*_max_‐values of 530 nm (Figure 1). This was accompanied with hyperchromic shifts (i.e., increase in absorbance) by 20% (from Abs 1.0 to 1.25) at pH 2, 30.5% (from Abs 0.8 to 1.15) at pH 3, and 51% (from Abs 0.51 to 1.0) at pH 4 (Figure 1). At pH 6, the addition of SDS led to a 6‐nm hypsochromic shift from *λ*_max_‐value of 546 to 540 nm, and almost the same absorbance was observed (Abs 0.41 without SDS and Abs 0.42 with SDS). However, due to the generally broader absorption peaks at this pH, the relative impact of this spectral shift is reduced.

Overall, the results indicated that the addition of SDS protected the anthocyanins from deprotonation reactions during pH changes between pH 2 and 4 (Figure 1). This could also explain why a hypsochromic shift occurred only at pH 6. Normally, the maximum absorbance shifts to higher wavelengths with the addition of SDS, but when the anthocyanin molecules are shielded from pH changes by SDS, it results in a decrease in the maximum wavelength at higher pH values, such as pH 6. Furthermore, the presence of SDS led to hyperchromic shifts upon increasing the pH (Figure 1). The stabilization of anthocyanins is attainable through a micellar system such as formed by the SDS molecules by the arrangement of their polar heads on the surface and through the ionic interaction between the positively charged flavylium cations and the negatively charged SDS molecules (Mulinacci et al. 2001). No SDS micelles, however, were formed in our system as the concentration of SDS at 3.47 mM (i.e., 0.1%) was below the critical micelle concentration of 4.61 mM (Fuguet et al. 2005). Stabilization by SDS below the critical micelle concentration may involve electrostatic interactions between the flavylium cation and the SDS. Similar effects have been described between anthocyanins and polyelectrolytes, such as various polysaccharides and proteins (Tan et al. 2021), where co‐pigmentation reactions protect the flavylium cation from nucleophilic attack by water, resulting in better stability against increasing pH (Enaru et al. 2021). However, further techniques such as Fourier transform infrared spectroscopy or isothermal titration calorimetry should be employed to prove such stabilization mechanisms between SDS and flavylium cations in future studies.

### 3.3 Lipid Oxidation in Emulsion

The purpose of the lipid oxidation analyses was to examine the influence of pH and the antioxidant effect of black carrot extract on the formation of oxidation products in the flaxseed oil‐in‐water emulsions during 21 days of storage at 35°C in the dark.

**FIGURE 2:**
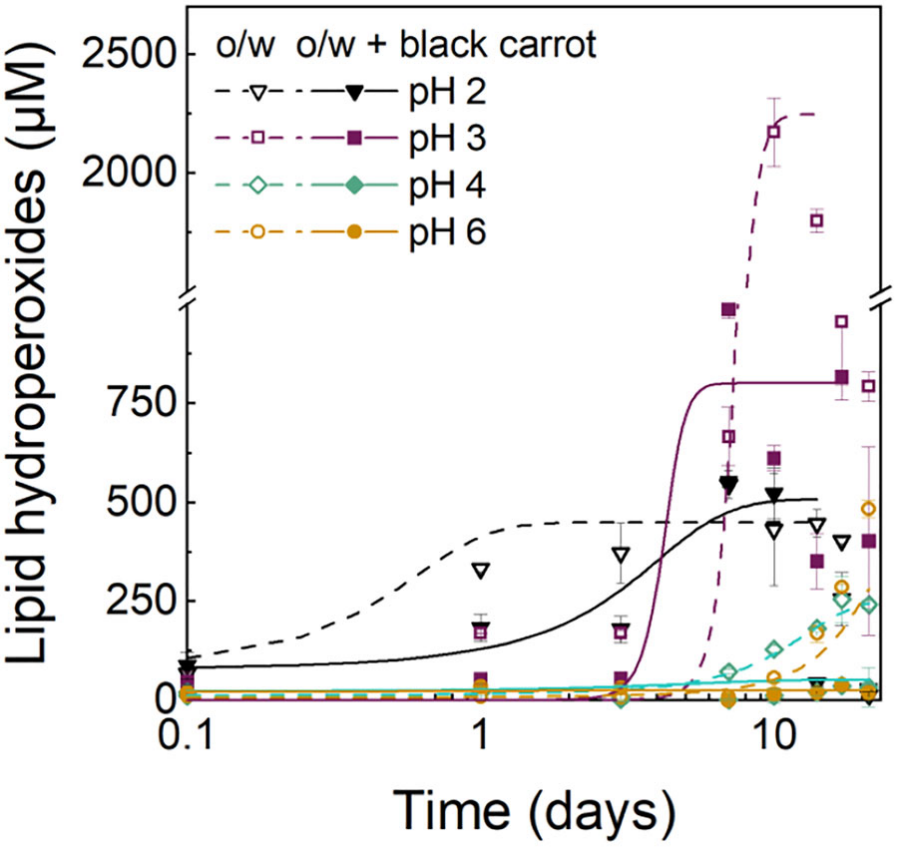
Formation of lipid hydroperoxides in flaxseed oil‐in‐water emulsions (1% w/w oil, 0.1% w/w SDS in 10 mM citrate buffer) at different pH values with or without added black carrot extract (0.73 g L^−1^) during 21 days of storage at 35°C in the dark. The lines shown represent sigmoidal data fittings.

**FIGURE 3:**
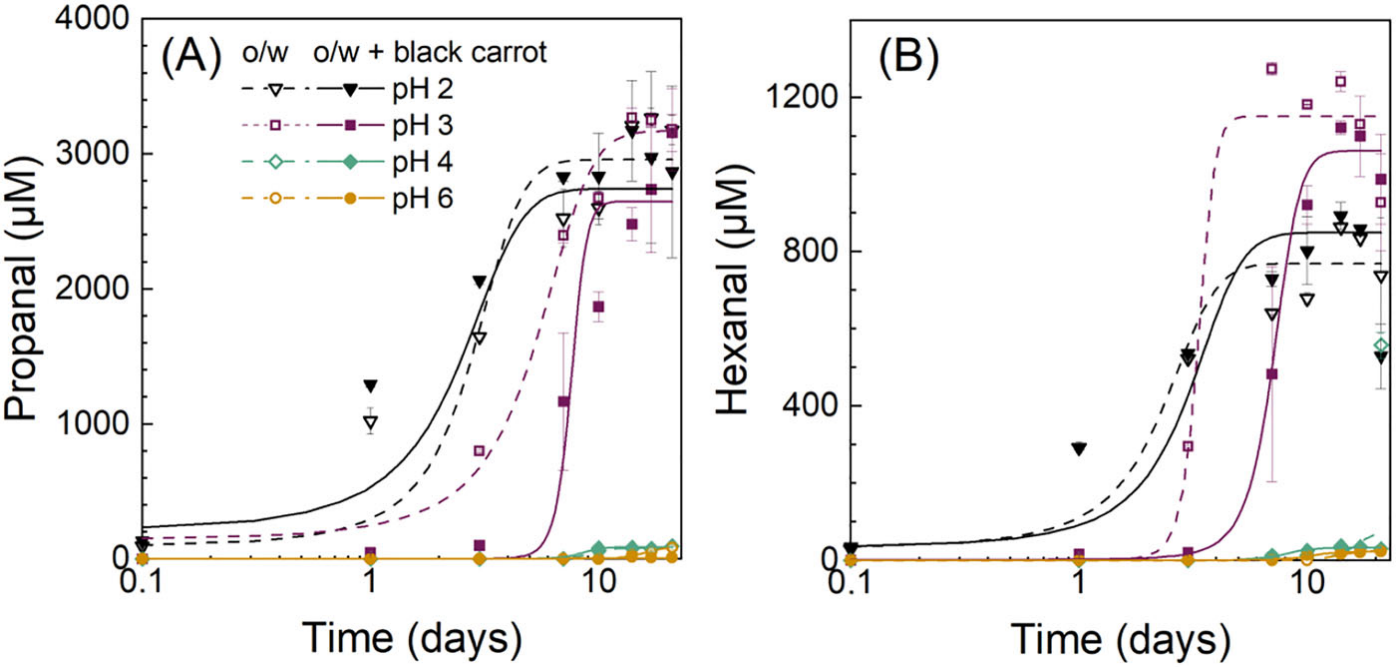
Formation of propanal (A) and hexanal (B) in flaxseed oil‐in‐water emulsions (1% w/w oil, 0.1% w/w SDS in 10 mM citrate buffer) at different pH values with or without added black carrot extract (0.73 g L^−1^) during 21 days of storage at 35°C in the dark. The lines shown represent sigmoidal data fittings. The legend applies to both figures.

The data showed that both pH and the addition of black carrot extract affected the formation of both primary (Figure 2) and secondary oxidation products (Figure 3). To gain more insights into the kinetic behavior upon oxidation, the induction period (*t*_ind_) and rate constant *k* were calculated (Table 2). The rate constant *k* indicates the tendency to generate free radicals during the initiation phase.

**TABLE 2:** Kinetic parameters^a^ of primary and secondary lipid oxidation products formed in flaxseed oil‐in‐water emulsions (1% w/w oil, 0.1% w/w SDS in 10 mM citrate buffer) at different pH values with or without added black carrot extract during storage at 35°C in the dark.

Oxidation product	pH	*c*_extract_ (g L^−1^)	*t*_ind_ (days)^b^	*k* (day^−1^)^c^
LOOH	2	0	—	4.0 ± 1.6d
		0.73	—	1.9 ± 0.1c
	3	0	6.3 ± 0.1ab	1.6 ± 0.1bc
		0.73	5.7 ± 2ab	0.9 ± 0.24ab
	4	0	2.9 ± 1.5b	0.3 ± 0.04a
		0.73	8.8 ± 2.9b	0.5 ± 0.2a
	6	0	9.1 ± 0.8b	0.2 ± 0.06a
		0.73	20.8 ± 7.4c	0.1 ± 0a
Propanal	2	0	1.1 ± 0.1a	1.3 ± 0.1ab
		0.73	0.8 ± 0.2a	1.8 ± 0.9b
	3	0	1.8 ± 0.04a	0.6 ± 0.02a
		0.73	6.5 ± 1.3b	1.6 ± 0.3b
	4	0	7.3 ± 0.5b	1.0 ± 0.1ab
		0.73	6.6 ± 0.7b	1.1 ± 0.1ab
	6	0	11.8 ± 1.2c	0.6 ± 0.1a
		0.73	10.8 ± 2.8c	0.5 ± 0.1a
Hexanal	2	0	0.8 ± 0.1a	1.4 ± 0.1b
		0.73	1.3 ± 0.6a	1.0 ± 0.03ab
	3	0	2.7 ± 0.02ab	1.0 ± 0.02c
		0.73	5.2 ± 1bc	1.0 ± 0.01ab
	4	0	12.4 ± 1d	0.9 ± 0.02a
		0.73	5.9 ± 0.3c	1.0 ± 0.01ab
	6	0	10 ± 1.3d^e^	1.0 ± 0.01a
		0.73	7.9 ± 1.9c^e^	0.9 ± 0.06a

*Note*: Different small letters within each oxidation product and kinetic parameter denote a significant difference according to Tukey post hoc test (*p* < 0.05).

Abbreviation: LOOH, lipid hydroperoxides.

^a^ The values were calculated from sigmoidal functions fitted on the kinetic curves (Figures 2 and 3).

^b^ Induction time *t*_ind_ was calculated according to the inflection tangent reaching *c* = 0 µM; —, no induction phase could be calculated.

^c^ Rate constant *k* was calculated according to Equation (3).

The flaxseed oil‐in‐water emulsions at pH 2 with or without added black carrot extract showed initially (i.e., on the day of the sample preparation) slightly higher concentrations of lipid hydroperoxides (67 µM, *p* < 0.05) than the emulsions at pH 3 (29 µM), pH 4 (21 µM), and pH 6 (12 µM) (*p* > 0.05) (Figure 2). The lower the pH, the faster the lipid hydroperoxides formed (Figure 2), as also demonstrated by generally shorter induction periods and faster rate constants at lower pH (Table 2). However, the formation rate appeared to approach a plateau, as no statistically significant differences were observed between pH 4 and 6 for the rate constant *k* (*p* > 0.05), nor for the concentration of lipid hydroperoxides at the end of the storage period (without black carrot extract: pH 4 = 255 ± 59 µM; pH 6 = 286 ± 8 µM). This is similar to a previous study that showed an increased formation of primary oxidation products with decreasing pH in SDS‐stabilized oil‐in‐water emulsions (Mei, McClements, et al. 1998). Similarly, Kim and Choe (2018) found higher lipid hydroperoxide concentrations in oil‐in‐water emulsions at pH 2.6 compared to pH 4 or 6 due to the higher solubility of Fe^2+^ at acidic pH inducing iron‐catalyzed lipid oxidation. On the other hand, a study by Sui et al. (2014) reported that the antioxidant activity of anthocyanins was not influenced by pH in aqueous buffer solution at pH 2.2–6.0. Furthermore, a study by Li et al. (2020) reported a decrease in the primary oxidation products upon increasing the pH from 3 to 7 in *Quillaja* saponin‐stabilized flaxseed oil‐in‐water emulsions containing tannic acid. The authors explained this by a possibly reduced antioxidant activity of polyphenols, a reduced metal‐chelating capacity of chelators like citric acid, and also an increased solubility of prooxidant transition metals.

The addition of black carrot extract also slowed down the formation of lipid hydroperoxides in the flaxseed oil‐in‐water emulsions (Figure 2). At pH 2, the black carrot extract slowed down the formation of lipid hydroperoxides during the early days of oxidation (*p* < 0.05) until reaching a plateau after 7 days (Figure 2). At pH 3, less lipid hydroperoxides (*p* < 0.05) were formed in the emulsions with the black carrot extract (*c*_max_ = 986 ± 23 µM) than in the one without the extract (*c*_max_ = 2172 ± 144 µM), which was also illustrated by the slower *k*‐values (*p* < 0.05) with the added extract (Table 2). At pH 4 and 6, the addition of the black carrot extract slowed down the formation of lipid hydroperoxides substantially, as their concentrations remained low (≤ 40 µM) during the 21 days of storage, whereas the emulsions without the extract generated more lipid hydroperoxides at both pH 4 (*c*_max_ = 255 ± 59 µM) after 17 days and at pH 6 (*c*_max_ = 484 ± 22 µM) after 21 days (Figure 2). These results are consistent with previous studies reporting that anthocyanins from black carrots (pH 2) (Klinger et al. 2024a), black rice (pH 7) (Yi et al. 2020), purple sweet potato (pH 7) (Gan et al. 2012), and black currant (pH 7) (Salminen et al. 2010) reduced lipid hydroperoxide formation in oil‐in‐water emulsions.

The further degradation of the lipid hydroperoxides led to the formation of volatile secondary oxidation products, such as propanal and hexanal (Figure 3), which are markers of the oxidation of ω‐3 and ω‐6 fatty acids, respectively (Frankel 2014). In the emulsions without added black carrot extract at pH 2, small concentrations of both propanal (93 ± 15 µM) and hexanal (29 ± 4 µM) were already detected directly after the emulsion preparation, whereas no secondary oxidation products were detected at pH 3, 4, and 6 (Figure 3). Similar to primary oxidation products, the formation of propanal and hexanal was dependent on the pH and occurred faster with decreasing pH (Figure 3). This can be also illustrated by the shorter induction periods at lower pH values (Table 2). The induction periods in forming propanal increased in the order of pH 2 ≅ pH 3 > pH 4 > pH 6 for the flaxseed oil‐in‐water emulsions. Similarly, the induction periods in forming hexanal increased in the order of pH 2 ≅ pH 3 > pH 4 ≅ pH 6 (*p* < 0.05). Nevertheless, the flaxseed oil‐in‐water emulsions at pH 3 oxidized the most as demonstrated by the highest propanal (*c*_max_ = 3270 ± 70 µM) and hexanal concentrations (*c*_max_ = 1241 ± 26 µM) measured during the storage time (Figure 3; Table S1). The higher hexanal formation observed at pH 3 compared to pH 2 may be related to the reaction pathways of lipid hydroperoxides under highly acidic conditions. The highly acidic environment, particularly in the presence of trace metal ions, may inhibit the cleavage of lipid hydroperoxides into volatile aldehydes such as hexanal. Instead, hydroperoxides can undergo alternative acid‐catalyzed rearrangements or decompositions that do not lead to hexanal formation (Frankel 2014). For lipid hydroperoxides, no differences (*p* > 0.05) in rate constant *k* for propanal and hexanal formation were observed between pH 4 and 6 (Table 2).

At pH 2, the addition of black carrot extract showed a prooxidant effect in forming propanal during the first 3 days of storage (*p* < 0.05); however, no statistical differences between the emulsions with and without black carrot extract were found at later stages of oxidation (Figure 3). No statistical differences were found in hexanal formation between the emulsions with and without black carrot extract (Figure 3).

At pH 3, the addition of black carrot extract into the flaxseed oil‐in‐water emulsion inhibited the formation of propanal (*p* < 0.05) (Figure 3) as was also indicated by the longer induction period (*p* < 0.05) and faster rate constants (*p* < 0.05) (Table 2). The black carrot extract also inhibited the formation of hexanal slightly during the propagation phase, showing lower hexanal concentrations (*p* < 0.05) at pH 3 (Figure 3). No statistical differences were, however, found for the *t*_ind_‐values between the emulsions with and without extract (Table 2).

**FIGURE 4:**
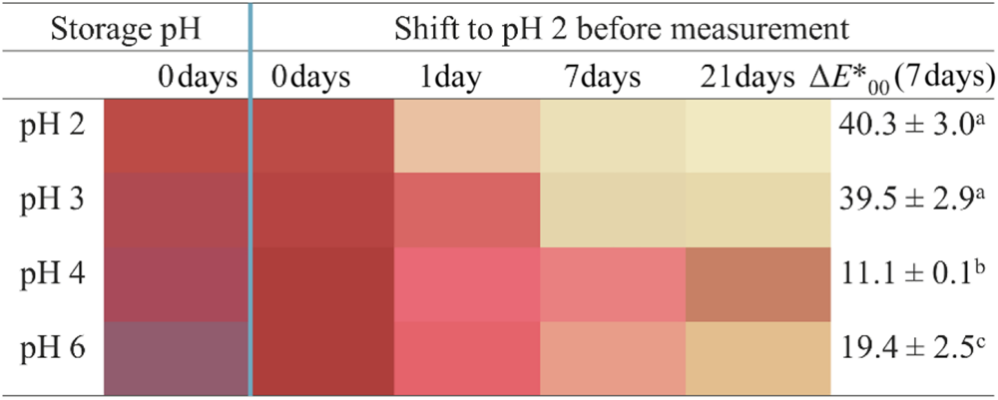
Colors of flaxseed oil‐in‐water emulsions (1% w/w oil, 0.1% w/w SDS in 10 mM citrate buffer) at different pH values with added black carrot extract (0.73 g L^−1^) during 21 days of storage at 35°C in the dark. During storage, the color was measured after a shift to pH 2. Color differences ($\Delta E_{00}^{*}$) were calculated between 0 and 7 days.

At pH 4, the concentration of both propanal and hexanal remained very low (≤86 µM) throughout the storage period, indicating minimal lipid oxidation. However, at the final point (Day 21), slightly lower concentrations of both aldehydes were observed in emulsions containing black carrot extract compared to the control, suggesting a potential onset of antioxidant activity (Figure 4). Due to the overall low extent of oxidation, this observation should be interpreted with caution. At pH 6, the addition of the extract had no impact on the oxidation of the flaxseed oil‐in‐water emulsions. It should be noted that the hexanal detected at pH 6 was also at a low concentration level (≤82 µM) for all samples.

Overall, the higher the pH, the slower the formation of primary and secondary oxidation products. This is in line with previous studies on lipid oxidation at different pH values as well as antioxidant activity of anthocyanins (Mei, McClements, et al. 1998; Kim and Choe 2018; Li et al. 2020; Yi et al. 2020). Li et al. (2020) observed an increase in lipid oxidation with decreasing pH in saponin‐coated 5% flaxseed oil‐in‐water emulsions (pH 3.5 and 7), attributed to the enhanced solubility and activity of ferrous ions in acidic environments. In a previous study, the total amount of iron in the black carrot extract was found to be 0.79 mM (Klinger et al. 2024a). Based on a concentration of 0.73 g L^−1^ black carrot extract used in the emulsions, this corresponds to an iron concentration of approximately 0.58 µM. Given the catalytic role of transition metals such as Fe^2^⁺ in initiating and propagating lipid oxidation, the presence of iron originating from the extract may have contributed to the oxidative processes observed. A similar effect was reported by Mei, McClements, et al. (1998) in corn oil‐in‐water emulsions stabilized by SDS (pH 3–8). Kim and Choe (2018) found higher lipid oxidation at pH 4 compared to pH 2.6 and 6, with the best antioxidant activity of peppermint extract polyphenols observed at pH 6 in soybean oil‐in‐water emulsions stabilized by xanthan gum and lecithin. This was explained by the greater stability of peppermint polyphenols (primarily rosmarinic acid, caffeic acid, and luteolin) at pH 6 (Kim and Choe 2018). Similarly, Zhou and Elias (2013) reported increased lipid oxidation with decreasing pH (pH 2–7) in Tween 80‐stabilized 10% flaxseed oil‐in‐water emulsions, which they attributed to the higher solubility and activity of metal ions catalyzing the reaction. Additionally, a prooxidant effect of epigallocatechin‐3‐gallate was observed at pH values below 4, likely due to competing reactions between reactive oxygen species generation associated with metal‐catalyzed polyphenol oxidation and the radical‐scavenging activity of polyphenols (Zhou and Elias 2013).

### 3.4 Color and Anthocyanin Degradation of Black Carrot Extract in Emulsions

The red color of the black carrot extract in the flaxseed‐oil‐in‐water emulsions at pH 2 changed to increasing hues of purple upon increasing the pH to 6 (Figure 4). This is due to the structural changes in their molecular form (Section 3.2 ). All emulsions at pH 2–6 showed a red color directly after preparation (upon shift to pH 2); however, the color faded from red over pink to light beige upon storage for 21 days. The loss of red color can be attributed to the delocalization of electrons in the anthocyanin ring structure due to the donation of hydrogen to a lipid peroxide radical while acting as a radical scavenger (Dangles and Fenger 2018).

**FIGURE 5:**
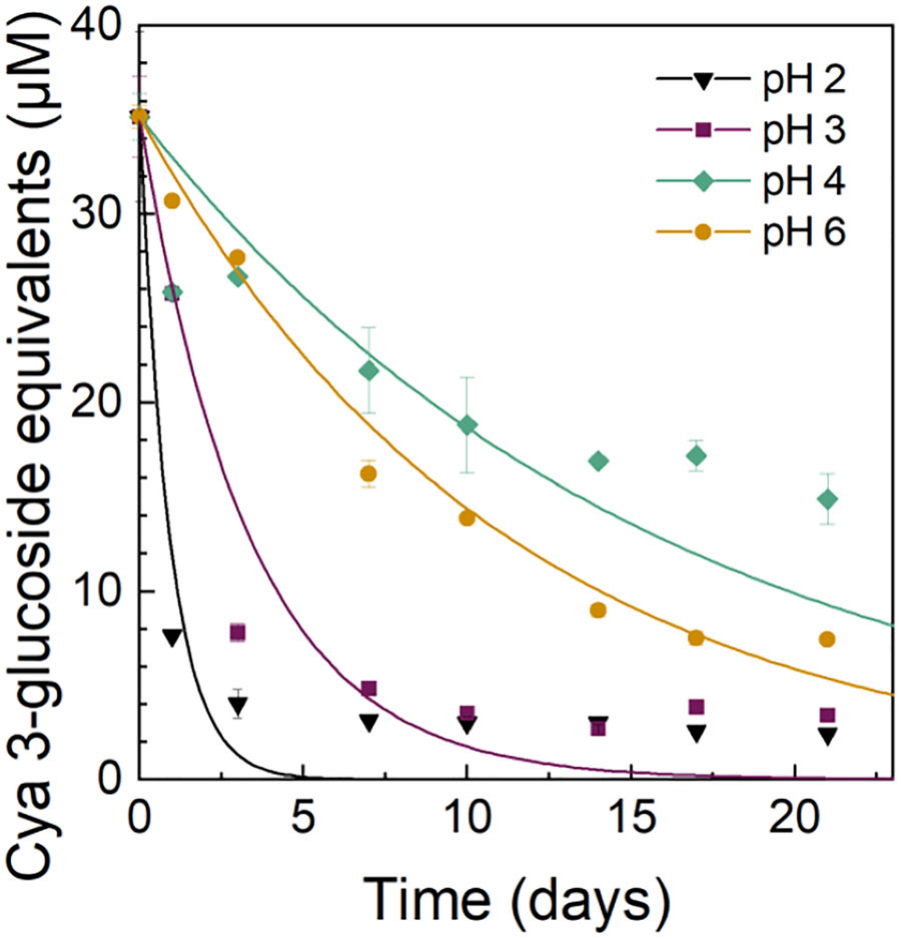
Degradation of black carrot anthocyanins as cyanidin 3‐glucoside equivalents in flaxseed oil‐in‐water emulsions (1% w/w oil, 0.1% w/w SDS in 10 mM citrate buffer) at different pH values with added black carrot extract (0.73 g L^−1^) during 21 days of storage at 35°C in the dark after hexane extraction.

The kinetics and the extent of the color loss depended on the pH of the emulsion (Figure 4). At pH 2, the red color of the emulsion faded almost completely after only 1 day of storage (Figures 4 and 5). This was also denoted by the fastest reaction rate *k* (*p* < 0.05) and shortest half‐time life *t*_1/2_ upon anthocyanin degradation (Table 3). The complete color loss occurred after 7 days (Figures 4 and 5), illustrated by the increased lightness (L* = from 53 ± 7 to 90 ± 0.4), decreased redness (a* = from 46 ± 11 to −0.3 ± 0.3), and slightly decreased yellowness (b* = from 25 ± 3 to 21 ± 1) upon 7 days of storage. Similar color degradation and degradation kinetics (*k* = 2.5 day^−1^; *t*_1/2_ = 0.5 days) of black carrot extract in flaxseed oil‐in‐water emulsions at pH 2 were observed in our earlier study (Klinger et al. 2024a).

**TABLE 3:** Kinetic parameters^a^ of anthocyanin degradation in flaxseed oil‐in‐water emulsions (1% w/w oil, 0.1% w/w SDS in 10 mM citrate buffer) at different pH values with added black carrot extract (0.73 g L^−1^).

pH	*k* (day^−1^)^b^	*t*_1/2_ (days)^c^
2	1.44 ± 0.01c	0.48 ± 0.00a
3	0.30 ± 0.17b	1.81 ± 0.06a
4	0.06 ± 0.01a	11.13 ± 1.81b
6	0.09 ± 0.03a	8.53 ± 3.55b

*Note*: Different small letters within each kinetic parameter denotes a significant difference according to Tukey post hoc test (*p* < 0.05).

^a^ The values were calculated from exponential functions fitted on the kinetic curves (Figure 5).

^b^ Rate constant *k* was calculated according to Equation (1).

^c^ Half‐life time *t*_1/2_ was calculated according to Equation (2).

At pH 3, the red color degraded slightly slower compared to pH 2 (Figures 4 and 5) as demonstrated by a slower *k*‐value (*p* < 0.05) and a longer *t*_1/2_‐value (*p* > 0.05) (Table 3). The red color was still observed on day 1, after which it faded considerably after 3 days, and was fully lost after 7 days of storage (Figures 4 and 5). This was indicated by the changes in color attributes such as increased lightness (L* = from 46 ± 4 to 80 ± 0.4), decreased redness (a* = from 47 ± 2 to 3 ± 0.3), and decreased yellowness (b* = from 27 ± 0.1 to 25 ± 0.5) upon 7 days of storage. This was similar to increased lightness (L*) and yellowness (b*) values observed in the color from black carrot anthocyanins in liposomes at pH 3.5, leading to yellow hue during storage (Guldiken et al. 2018).

At pH 4 and 6, the color of emulsions faded significantly (*p* < 0.05) slower during the 7 days of storage compared to emulsions at pH 2 and 3 as also indicated by the color difference values ΔE*_00_ (Figure 4) and the slower *k*‐values and longer *t*_1/2_‐values (Table 3). The emulsions at pH 4 showed the best color stability as some brownish color was still observed after 21 days of storage (Figure 5). The changes in lightness are typical for an irreversible degradation reaction of anthocyanins, whereas a slight yellow shift can be an indicator of polymerization reactions (Jiang et al. 2019).

To our knowledge, no kinetic parameters for the degradation of anthocyanins from black carrot extract at pH 3, 4, and 6 have been reported in the literature to date. Chen et al. (2020) reported a reaction rate *k* of 0.08 day^−1^ and a half‐life of 9.2 days for heat‐treated (95°C, 5 min) raspberry anthocyanins at pH 3 in a model juice containing 10% sucrose during storage at 37°C. Jiang et al. (2019) found that anthocyanins from purple sweet potato in citrate buffer (8 g L^−1^) stored at 90°C degraded the fastest at pH 7 (*k* = 3.57 day^−1^; *t*_1/2_ = 0.19 days), followed by a slightly slower degradation at pH 3 (*k* = 1.62 day^−1^; *t*_1/2_ = 0.43 days) and pH 5 (*k* = 1.34 day^−1^; *t*_1/2_ = 0.52 days). The differences were explained by different hydration equilibrium constants of anthocyanin forms at different pH values (Jiang et al. 2019; Stintzing et al. 2002). It should be also noted that the studied systems contained no (dispersed) lipids.

Overall, the black carrot anthocyanin stability in the flaxseed oil‐in‐water emulsions increased with increasing pH, similarly to the lipid oxidation products. Contrary to the common perception that anthocyanin stability decreases with increasing pH, in systems involving lipid oxidation, the radical‐scavenging reactions driven by the antioxidant activity of anthocyanins appear to outweigh the effects of pH changes caused by water addition (Castañeda‐Ovando et al. 2009; Dangles and Fenger 2018; Sui et al. 2014; Jiang et al. 2019; Stintzing et al. 2002). The color in oil‐in‐water emulsions at pH 6 degraded faster than at pH 4 (Figures 4 and 5), even though the extent of lipid oxidation was similar (Figures 2 and 3). This suggests that at a similar lipid oxidation rate, the stability of the anthocyanin molecule is again dictated by the deprotonation reactions. The deprotonation reactions upon increasing the pH have been shown to decrease the stability of anthocyanins (Stintzing et al. 2002; Sui et al. 2014; Jiang et al. 2019; Castañeda‐Ovando et al. 2009; Dangles and Fenger 2018).

The findings of this study provide practical implications for the formulation of food products based on oil‐in‐water emulsions such as red‐colored fruit yogurts and yogurt drinks in which lipid oxidation of the unsaturated fatty acids of milk fat plays a critical role (Citta et al. 2017) and anthocyanins show reduced stability even at pH values near their optimum (Ścibisz et al. 2019). Similar considerations apply to nondairy alternatives such as soy or oat in which lipid oxidation is also a major quality‐degrading factor (Moretto et al. 2021). In the confectionery sector, color is a key attribute contributing to product appeal, and thus, extended shelf life is of particular importance (Granados‐Balbuena et al. 2024). Cream fillings based on emulsions containing palm fat or rapeseed oil, commonly used in bakery products, have been shown to be particularly susceptible to lipid oxidation (Bełkowska et al. 2025). This degradation can be mitigated through the incorporation of polyphenolic compounds, such as anthocyanin‐rich black carrot extract. However, such interactions may lead to undesirable color changes, especially in fruit‐flavored products, where consumers associate high quality with bright and stable coloration.

### 3.5 Mechanistic Insights Into the Effects of pH and SDS on Anthocyanin Stability and Lipid Oxidation

The results of this study (Figures 1, 2, 3, 4, 5; Tables 1, 2, 3) showed that the radical reactions of anthocyanins in oxidatively unstable flaxseed oil‐in‐water emulsions took precedence over the well‐known pH‐induced deprotonation reaction.

We propose that SDS at a concentration below the CMC can stabilize anthocyanins against pH‐dependent deprotonation, similar to known copigments such as polysaccharides and proteins (Tan et al. 2021).

In terms of lipid oxidation in flaxseed oil‐in‐water emulsions, the extent of oxidation decreased with an increasing pH. This phenomenon may be explained by the higher solubility of catalyzing metal ions at lower pH as discussed in previous literature (Kim and Choe 2018). We hypothesized that the presence of trace metals, introduced by the black carrot extract, could have contributed to this effect.

The elevated lipid oxidation rate at acidic pH was associated with increased radical scavenging reactions of anthocyanins. This in turn led to accelerated anthocyanin degradation and color loss. Subsequently, the lowest color stability was observed at pH 2, followed by pH 3 and 6, whereas the best color stability was observed at 4. It seems that the radical scavenging reaction of the anthocyanins predominates over destabilization due to increasing pH.

At very low rates of lipid oxidation, the anthocyanin molecules undergo destabilization through deprotonation reactions as the pH increases from 4 to 6. This leads to a shift in the equilibrium from more stable flavylium cation at pH 2 and anhydrous quinoidal base at pH 4 toward the less stable carbinol pseudobase at pH 5 and chalcone at pH 6 (Castañeda‐Ovando et al. 2009).

## 4 Conclusion

This study revealed an intricate interaction between an anthocyanin‐rich black carrot extract and ω‐3 fatty acid–rich flaxseed oil‐in‐water emulsions and highlighted the central role of pH. The formation of both lipid hydroperoxides and volatiles was strongly influenced by the pH of the oil‐in‐water emulsion, showing increased lipid oxidation upon decreasing the pH. Correlating with the formation of lipid oxidation products, the stability of the anthocyanins from black carrot extract decreased as they acted as antioxidants. At similar rates of lipid oxidation, the stability of the anthocyanins decreased according to their pH‐induced deprotonation reactions. Therefore, the best color stability of the black carrot extract in the flaxseed oil‐in‐water emulsions was observed at pH 4. The results show how the common understanding about the stability of anthocyanin in aqueous solutions is limited when applied to emulsion‐based systems. It should be noted that even though SDS is an excellent surfactant in stabilizing oil‐in‐water emulsions and serves as a good choice for model systems, it is not suitable for most food applications. Therefore, more studies with other food‐grade emulsifiers are needed. Nevertheless, this study provides valuable mechanistic insights into the stability of anthocyanins in lipid‐containing systems.

## Author Contributions

**Evelyn Klinger**: conceptualization, investigation, formal analysis, visualization, writing – original draft. **Hanna Salminen**: conceptualization, visualization, writing – original draft, writing – review and editing, supervision. **Karola Bause**: conceptualization, writing – review and editing, supervision. **Jochen Weiss**: conceptualization, writing – review and editing, resources, supervision.

## Conflicts of Interest

The authors declare no conflicts of interest.

## Supporting information




**Supplementary Materials**: jfds70568‐sup‐0001‐SuppMat.docx
