# Supplementary material for: Effect of pH on Lipid Oxidation and Anthocyanin Stability in Flaxseed Oil‐in‐Water Emulsions With Black Carrot Extract
Source: J Food Sci. 2025 Sep 21;90(9):e70568. doi: 10.1111/1750-3841.70568 (PMC12451094; doi:10.1111/1750-3841.70568)
Supplement: Supplementary file 1 — Supplementary Materials: jfds70568‐sup‐0001‐SuppMat.docx [file JFDS-90-0-s001.docx]

**Supplemental Information**

Table S1 Sigmoidal data fits and coefficient of determination (R^2^) of primary and secondary oxidation products formed in flaxseed oil-in-water emulsions (1% *w/w* oil, 0.1% *w/w* SDS in 10 mM citrate buffer) at pH 2, 3, 4 and 6 without or with 0.73 g/L black carrot extract during storage at 35°C in the dark

| $c=\frac{c_{\max}}{1 +(\frac{c_{\max-c_{o}}}{c_{0}})\cdot e^{-kt}}$ | | Lipid hydroperoxides | | | Propanal | | | Hexanal | | | |
| --- | --- | --- | --- | --- | --- | --- | --- | --- | --- | --- | --- |
|  | *c*_extract_ (g/L) | *c*_max_ (µM) | *c*_0_ (µM) | R^2^ | *c*_max_ (µM) | *c*_0_ (µM) | R^2^ | *c*_max_ (µM) | *c*_0_ (µM) | | R^2^ |
| pH 2 | 0 | 450 ± 41 | 97 ± 53 | 0.77 ± 0.05 | 2957 ± 57 | 93 ± 15 | 0.9 ± 0.03 | 778 ± 1 | 122 ± 6 | 0.93 ± 0.02 | |
|  | 0.73 | 528 ± 52 | 71 ± 20 | 0.91 ± 0.08 | 2741 ± 407 | 142 ± 26 | 0.92 ± 0.06 | 849 ± 19 | 34 ± 1 | 0.95 ± 0.03 | |
| pH 3 | 0 | 2249 ± 20 | 0.01 ± 0.01 | 0.96 ± 0.03 | 3170 ± 87 | 144 ± 7 | 0.98 ± 0.01 | 1151 ± 29 | 0.01 ± 0.01 | 0.96 ± 0.02 | |
|  | 0.73 | 805 ± 233 | 0.01 ± 0.01 | 0.75 ± 0.11 | 2649 ± 189 | 0.01 ± 0 | 0.93 ± 0.03 | 1061 ± 79 | 1 ± 0.2 | 0.99 ± 0.01 | |
| pH 4 | 0 | 259 ± 23 | 15 ± 6 | 0.96 ± 0.04 | 79 ± 10 | 0.01 ± 0 | 0.97 ± 0.03 | 76 ± 6 | 0.01 ± 0.01 | 0.97 ± 0.02 | |
|  | 0.73 | 51 ± 27 | 0.1 ± 0 | 0.81 ± 0.2 | 88 ± 3 | 0.01 ± 0 | 0.92 ± 0.06 | 33 ± 3 | 0.01 ± 0.01 | 0.96 ± 0.01 | |
| pH 6 | 0 | 485 ± 18 | 10 ± 5 | 0.99 ± 0 | 85 ± 15 | 0.02 ± 0.01 | 0.97 ± 0.03 | 25 ± 5 | 0.01 ± 0.01 | 0.95 ± 0.01 | |
|  | 0.73 | 39 ± 6 | 10 ± 4 | 0.83 ± 0.1 | 10 ± 1 | 0.01 ± 0.01 | 0.88 ± 0.1 | 22 ± 3 | 0.01 ± 0.01 | 0.93 ± 0.06 | |

Table S2 Exponential data fits and coefficient of determination (R^2^) of anthocyanin degradation in flaxseed oil-in-water emulsions (1% w/w oil, 0.1% w/w SDS in 10 mM citrate buffer) and in aqueous SDS (0.1 % w/w) solutions at pH 2, 3, 4, and 6 with 0.73 g/L black carrot extract during storage at 35°C in the dark.

| Sample $C_{t}=C_{0}\cdot e^{-kt}$ | o/w | |
| --- | --- | --- |
|  | C_0_ (µM) | R^2^ |
| pH 2 | 35.2 ± 4.5 | 0.94 ± 0.01 |
| pH 3 | 35.2 ± 2.1 | 0.94 ± 0.00 |
| pH 4 | 35.2 ± 1.2 | 0.77 ± 0.00 |
| pH 6 | 35.2 ± 0.6 | 0.98 ± 0.00 |


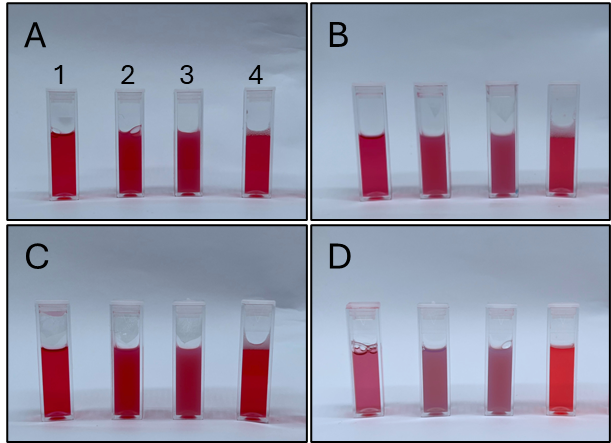


**Figure S1** Images of flaxseed oil-in-water emulsions (1% w/w oil, 0.1% w/w SDS in 10 mM citrate buffer) at pH 2 (A), pH 3 (B), pH 4 (C), and pH 6(D) with added black carrot extract (0.73 g/L) right after preparation. The cuvettes contain the following samples, from left to right: (1) aqueous phase of emulsion after hexane extraction at storage pH, (2,3) oil-in-water emulsion at storage pH, (4) aqueous phase of emulsion after pH shift to pH 2. This sample sequence applies to all pH values.


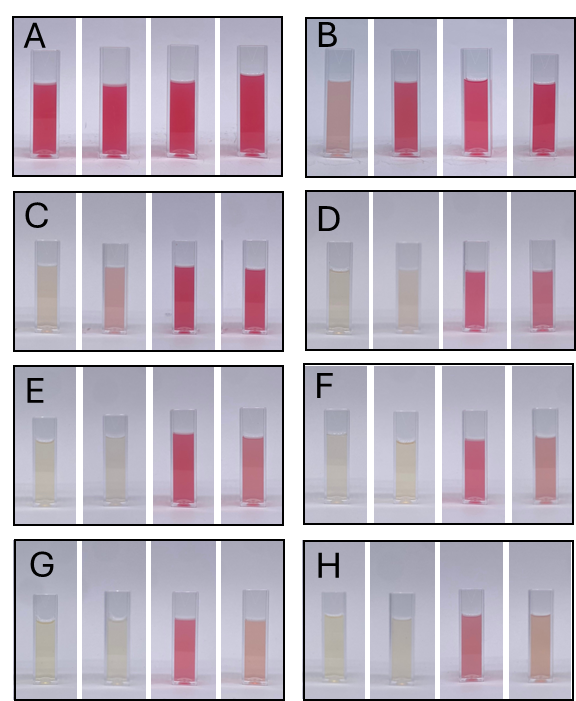


**Figure S2** Images of the aqueous phases of flaxseed oil-in-water emulsions (1% w/w oil, 0.1% w/w SDS in 10 mM citrate buffer) at different pH with added black carrot extract (0.73 g/L) during 21 days of storage at 35°C in the dark after hexane extraction. The cuvettes contain the following samples, from left to right: pH 2, pH 3, pH 4, pH 6 and the sample sequence applies to all images (A-H). Storage times: After (A) preparation (day 0), (B) day 1, (C) day 3, (D) day 7, (E) day 10, (F) day 14, (G) day 17, (H) day 21.
